# Supplementary material for: A National Accessibility Audit of Primary Health Care Facilities in Brazil—Are People with Disabilities Being Denied Their Right to Health?
Source: Int J Environ Res Public Health. 2021 Mar 13;18(6):2953. doi: 10.3390/ijerph18062953 (PMC7999795; doi:10.3390/ijerph18062953)
Supplement: Supplementary file 1 [file ijerph-18-02953-s001.pdf]

Supplementary 1

Title S1: Formula for calculation of total accessibility score

The total accessibility score was calculated by the following formulas:

$$TAS = \frac{\mathrm{x} - Min_x}{Max_x - Min_x} * 100 \ (1)$$

$$x = \frac{x_{i,a} - Min_a}{Max_a - Min_a} + \frac{x_{i,b} - Min_b}{Max_b - Min_b} + \frac{x_{i,c} - Min_c}{Max_c - Min_c} \ (2)$$

Where:

- TAS: Total Accessibility Score;

- $x_{i,a}$: sum of unit *i*´s dummies variables for unit external infrastructure dimension;

- $x_{i,b}$: sum of unit *i*´s dummies variables for unit's internal infrastructure dimension;

- $x_{i,c}$: sum of unit *i*´s dummies variables for dimension Accessibility features of primary care facilities for people with visual or hearing disabilities;

- Max_a: highest observed value of the sum of unit *i*´s dummies for the unit's external infrastructure dimension;

- Min_a: lowest observed sum value of unit *i*´s dummies variables for unit external infrastructure dimension;

- Max_b: highest observed value of the sum of unit *i*´s dummies for the unit's internal infrastructure dimension;

- Min_b: lowest observed sum value of unit *i*´s dummies variables for unit's internal infrastructure dimension

- Max_c: highest observed value of sum of unit *i*´s dummies for dimension Accessibility features of primary healthcare facilities for people with visual or hearing disabilities

- Min_c: lowest observed value of sum of unit *i*´s dummies for dimension Accessibility features of primary healthcare facilities for people with visual or hearing disabilities.
